# Metabonomics Combined with UPLC-MS Chemical Profile for Discovery of Antidepressant Ingredients of a Traditional Chinese Medicines Formula, Chaihu-Shu-Gan-San

**DOI:** 10.1155/2013/487158

**Published:** 2013-04-04

**Authors:** Hongmei Jia, Zhiheng Su, Wei Long, Yuetao Liu, Xing Chang, Hongwu Zhang, Gang Ding, Yufei Feng, Dayong Cai, Zhongmei Zou

**Affiliations:** ^1^Institute of Medicinal Plant Development, Chinese Academy of Medical Sciences and Peking Union Medical College, Beijing 100193, China; ^2^School of Pharmaceutical Science, Guangxi Medical University, Nanning 530021, China; ^3^Tianjin Key Laboratory of Molecular Nuclear Medicine, Institute of Radiation Medicine, Chinese Academy of Medical Science, Tianjin 300192, China; ^4^Department of Pharmacy, Beijing Hospital, Ministry of Public Health, Beijing 100730, China

## Abstract

This study proposed a new strategy for uncovering the active chemical constituents of a traditional Chinese medicines (TCMs) formula, Chaihu-Shu-Gan-San (CSGS). Metabonomics and chemical profile were integrated in combination with the multivariate statistical analysis (MVA) to discover the chemical constituents which contribute to the antidepressant effect of CSGS. Based upon the difference between CSGS and QZ (CSGS without Zhi-Qiao) extracts in the chemical profiles and the regulations of metabolic disturbances induced by CUMS, synephrine, naringin, hesperidin, and neohesperidin were recognized as the active constituents of CSGS from Zhi-qiao responsible for those missing regulations of CSGS when Zhi-Qiao was subtracted from the whole formula. They participated in the regulations of the deviated metabolites **2–4**, **10–14**, and **22–25**, involved in metabolic pathways of ketone bodies synthesis, phenylalanine, tyrosine and tryptophan biosynthesis, valine, aspartate, glutamate metabolism, and glycolysis/gluconeogenesis. Furthermore, the assay of MAO-A activity confirmed the potential antidepressant effect of naringin and its active sites on the MAO-A was inferred by molecular docking study. The integration of metabonomics and chemical profile was proved to be a useful strategy for uncovering what the active chemical constituents in TCM formula are and how they make contributions for the efficacy of the formula.

## 1. Introduction

Traditional Chinese medicines (TCMs) have been widely used in many oriental countries for thousands of years [1] and received widespread acceptance and attention due to their reliable therapeutic efficacy with low side effects [2]. It is universally acknowledged that the holistic and dynamic effects were achieved by multitargets interactions of the multiconstituents in TCMs. However, the complexity and interaction of multiconstituents in TCMs make the identification of the chemical constituents related to the efficacy and the definition of their mechanism of action challenging. Novel approaches are in great demand to provide deeper insight into the correlation of chemical constituents with efficacy of herbal formula. 

Chromatographic fingerprinting has been internationally accepted as an efficient technique for direct identification of multicomponents and quality control of TCMs [3]. Due to the wide suitability, high sensitivity, and sufficient structural information, liquid chromatography coupled with electrospray ionization tandem mass spectrometry (LC-ESI-MS^n^) has become more and more popular for investigation of herbal medicines [4]. Although on-line qualitative and quantitative analysis of chemical constituents in TCM formulas by LC-MS/MS was powerful for quality control, chromatographic profile fails to discern the correlation between the identified compounds and efficacy.

Metabonomics is the comparative analysis of metabolites and their dynamic flux associated with the response of living systems to pathophysiological stimuli or genetic modification [5]. Based on the global metabolic profile in biological samples such as urine, plasma, and tissue [2], it provides variation of the whole metabolic networks for characterizing pathological states in animals and humans, as well giving diagnostic information and presenting mechanistic insight into the biochemical effects of the toxins and drugs [6, 7]. In agreement with the holistic thinking of TCM, metabonomics has shown potential in evaluation of therapeutic effect of TCMs [8] and may provide the links needed for the complex metabolite mixtures in TCMs and molecular pharmacology [9].

Chaihu-Shu-Gan-San (CSGS) is one of the most widely used TCM formulas for treatment of depression clinically in China [10]. It is composed of seven Chinese herb medicines, that is, the roots of *Bupleurum chinense* DC (Chai-Hu), the pulp of *Citrus reticulata* Blanco (Chen-Pi), the roots of *Paeonia lactiflora* Pall (Bai-Shao), the pericarp of *Citrus aurantium* L. (Zhi-Qiao), the roots of *Cyperus rotundus* L. (Xiang-Fu), the roots of* Ligusticum chuanxiong* Hort (Chuan-Xiong), and the roots of* Glycyrrhiza uralensis* Fisch (Gan-Cao). The metabonomics study suggested that the antidepressant effect of CSGS could involve in regulating the dysfunctions of multiple metabolic pathways [11]. And the chemical constituents in CSGS were identified by LC-MS/MS and its antioxidant constituents were profiled by combination of 96-well plate collection of elutes from HPLC analysis and microplate spectrophotometer [12]. However, the contributions of chemical constituents in CSGS to its antidepressant effect are still not clear. 

**Figure 1:**
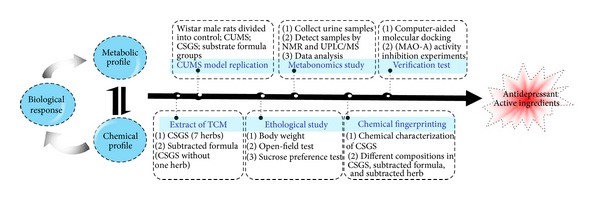
The proposed strategy for discovering the active constituents of CSGS responsible for its therapeutic effect on depression.

Here, a new integrated strategy of metabonomics and chemical profile in combination with the multivariate statistical analysis (MVA) was proposed to discover which of the chemical constituents in CSGS were responsible for its therapeutic effect (Figure 1). One of the key herbs in CSGS, Zhi-Qiao, was used as an example to explore the antidepressant chemical constituents from single herb in CSGS. The regulations of CSGS and QZ (CSGS without Zhi-Qiao) in metabolic disturbance induced by chronic unpredicted mild stress (CUMS) were explored by NMR and LC-MS-based metabonomics. Those regulations missed in QZ treated group should be related to the constituents existing in Zhi-Qiao but missed in QZ compared with CSGS. The chemical profiles of CSGS and QZ extracts through LC-MS/MS analysis were subjected to multivariate statistics analysis and those constituents that made contributions to discriminate the two extracts were considered as potential active constituents of CSGS from Zhi-Qiao, responsible for those effects that disappeared in QZ. Finally, combination of the results from metabonomics and chemical profile could tell us the contributions of Zhi-Qiao to CSGS both in chemical constituents and in regulations of metabolic pathways.

## 2. Materials and Methods

### 2.1. Chemicals and Reagents

HPLC-grade acetonitrile was purchased from Merck (Darmstadt, Germany). The water used for UPLC was purified by a Milli-Q system (Millipore, France). Formic acid (HPLC grade) was purchased from Tedia (Fairfield, USA). Pargyline, sodium phosphate, and sucrose were purchased from Sigma-Aldrich (St. Louis, MO, USA). Commercial kits used for determining MAO activity were obtained from the Jiancheng Institute of Biotechnology (Nanjing, China). Synephrine, hesperidin, neohesperidin and naringin were purchased from Tongtian Biotechnology Co., Ltd. (Shanghai, China). Fluoxetine hydrochloride was purchased from Eli Lilly and Company (Suzhou, China).

All raw herbal medicines were purchased from Beijing Tongren Tang Pharmaceutical Co. Ltd. (Beijing, China) and kept in our laboratory at Institute of Medicinal Plant Development, Chinese Academy of Medical Sciences, and Peking Union Medical College, China. The mixed crude herbs, Chai-Hu, Chen-Pi, Bai-Shao, Zhi-Qiao, Xiang-Fu, Chuan-Xiong, and Gan-Cao in the proportions of 4 : 4 : 3 : 3 : 3 : 3 : 1 by weight, were crushed into small pieces. The CSGS extract was prepared based on the traditional method as previously described [10]. The yield of the CSGS extract was 18.87%. The Zhi-Qiao (yield: 20.28%) and QZ (all herbs except Zhi-Qiao, yield: 20.00%) were prepared using procedures identical to that for CSGS.

### 2.2. Rats and Treatment

40 healthy, adult, male Wistar rats, weighing 200 ± 20 g each, were purchased from the Institute of Laboratory Animal Science, CAMS and PUMC (Beijing, China). The rats were housed individually in cages for one week to adapt to the environment under controlled conditions of 12 h light–12 h dark cycles (lights on from 6:00 a.m. to 6:00 p.m.), 10% relative humidity, and temperature (20 ± 3°C) with commercial diet and water available *ad libitum*. All experimental procedures were approved by the Ethics Committee of the Institute of Medicinal Plant Development, CAMS & PUMC.

The animals were randomly divided into 5 groups: (1) control group, (2) CUMS group, (3) positive control group, (4) CSGS treated group, and (5) QZ treated group. All rats except control group were subjected to a series of variable stimuli as previously described [10] after minor modification. The rats in the positive control, CSGS, and QZ treated groups were administrated with fluoxetine (3.0 mg/kg) and extracts of CSGS (equivalent to 31.5 g crude drug/kg body weight) and QZ (equivalent to 27.0 g crude drug/kg body weight), respectively, for 28 consecutive days, and the rats were sacrificed on the 28th day.

### 2.3. Sample Collection and Preparation 

All rats were housed in metabolic cages (1 per cage) so that the 24 h urine samples could be collected in collection bottles containing NaN_3_ (0.05% wt/vol) on the 28th day. 16 urine samples were randomly selected from the urine samples of each group, and 1 mL urine was taken from each sample, respectively; then the 16 mL urine was mixed together as the QC sample. The QC sample was used for the optimization of UPLC-Q-TOF/MS conditions. Every day, the stability of the instrument was tested with QC sample in order to make sure that the instrument was in the same condition during the whole analytical procedure. The urine samples were centrifuged (5000 rpm for 10 min, 4°C), and the supernatants were divided into two aliquots and stored at −80°C before analysis. One aliquot was used for NMR analysis and the other for UPLC/MS analysis. 

### 2.4. ^1^H NMR Spectroscopic Measurement of Urine Samples

An aliquot of 400 *μ*L urine was thawed at room temperature and mixed with 200 *μ*L of phosphate buffer (0.2 M Na_2_HPO_4_ and 0.2 M NaH_2_PO_4_ in D_2_O containing 0.05% wt/vol 3-trimethylsilyl-(2,2,3,3-^2^H_4_)-1-propionate (TSP); pH 7.4). Phosphate buffer minimized chemical shift variation because of different pH in urine samples, with D_2_O as a field lock and TSP as a chemical shift reference. The mixture was centrifuged (13000 rpm, 15 min) and the supernatant (550 *μ*L) of each sample was then transferred into a 5 mm o.d. NMR tube individually. All ^1^H NMR spectra were recorded at 300 K on a Bruker AV III 600 spectrometer (Bruker Biospin, Germany) equipped with an inverse 5 mm Bruker probe operating at 600.13 MHz ^1^H frequency. ^1^H NMR spectra were acquired using water-suppressed NOSEYGPPR1D (RD-90-*t*-90-*t*_*m*_-90-ACQ). The water signal suppression was achieved with weak irradiation on the water peak during the recycling delay (RD = 4.0 s) and mixing time (*t*_*m*_ = 0.10 s). The 90° pulse length was adjusted to *∼*10 *μ*s; a total of 128 transients were collected into 96 K data points over a spectral width of 20 ppm with an acquisition time of 3.07 s.

Prior to Fourier transformation, the FIDs for one-dimensional data were multiplied by an exponential function equivalent to a line broadening factor of 0.5 Hz and were zero-filled to 128 k. All NMR spectra were then corrected for phase and baseline distortions using Topspin software (v2.1, Bruker-Biospin, Germany). ^1^H NMR chemical shifts in the spectra were referenced to TSP at **δ** 0.00. All ^1^H NMR spectra from urine samples were data-reduced to integrated regions 0.004 ppm wide corresponding to the region **δ** 0.5–9.5 using AMIX software package (v3.9.2, Bruker Biospin, Germany). The regions **δ** 4.67–5.10 were removed to avoid the effect of residual water saturation. Each NMR data set was binned to 1875 variables to minimize the effects of pH and ionic concentrations.

### 2.5. UPLC/MS Measurement of Urine Samples

All urine samples were thawed at room temperature before analysis and centrifuged at 13,000 rpm for 10 min at 4°C. The supernatant was diluted at a ratio of 1 : 1 with water and an aliquot of 5 *μ*L was injected for UPLC-Q-TOF/MS analysis after filtration through a 0.22 *μ*M membrane filter.

The urine samples were analyzed on Waters Acquity Ultra Performance LC system (Waters Corporation, Milford, MA, USA) equipped with a BEH C18 column (100 mm × 2.1 mm, 1.7 *μ*m). The mobile phase was composed of water (A) and acetonitrile (B) each containing 0.1% formic acid. A solvent gradient system was used for detecting the urine samples: 1% B from 0 to 1 min, 1–32% B from 1 to 9 min, 32–99% B from 9 to 11 min, and 99% B from 12–15 min. The flow rate was 0.45 mL/min. All the samples were kept at 4°C during the analysis. The mass spectrometric data were collected using Q-TOF analyzer in SYNAPT HDMS system (Waters Corporation, Milford, MA, USA) in both positive and negative ion modes. Experimental parameters were set as previously described [11].

The raw data were analyzed using the MarkerLynx Applications Manager version 4.1 (Waters, Manchester, UK), which allowed deconvolution, alignment, and data reduction to give a list of retention time and mass pairs with corresponding intensities for all the detected peaks from each data file in the data set. The main parameters were set as follows: retention time range (RT) range 0.5–15 min, mass range 50–1200 amu, mass tolerance 0.02, minimum intensity 1%, mass window 0.05, retention time window 0.20, and noise elimination level 6.

The extracted ion chromatographic peaks of ten ions in positive mode (RT_*m*/*z*: 1.31_98.0603, 1.47_297.1443, 1.73_126.0919, 3.01_105.0334, 3.17_372.2382, 3.68_91.0541, 4.43_130.0654, 5.67_170.0612, 5.93_243.1021 and 6.68_203.1108) and ten ions in negative mode (RT_*m*/*z*: 0.83_182.0461, 1.23_144.0665, 2.21_158.0828, 3.00_178.0511, 3.38_222.0793, 4.31_283.0832, 4.76_173.0824, 6.10_187.0972, 7.36_201.1133 and 7.86_319.1380) from QC sample were selected for method validation. The repeatability of method was evaluated using six replicates of QC sample in positive and negative ion modes, respectively. The relative standard deviations (R.S.D%) of retention times and  *m*/*z*  were 0–0.4818% and 0.0002–0.0006% in positive mode and 0–0.3753% and 0.0003–0.0008% in negative mode, respectively. Precision of injection was carried out by six replicated analyses of the same urine sample. The relative standard deviations (R.S.D%) of retention times and  *m*/*z*  were 0–0.3719% and 0.0002–0.0007% in positive mode and 0.09–0.4173% and 0.0002–0.0009% in negative mode, respectively.

### 2.6. UPLC-MS/MS Analysis of the Extracts of CSGS, QZ, and Zhi-Qiao

50 mg of CSGS extract (equivalent to 264.97 mg of raw herbs in the proportions listed above) was dissolved in 5 mL of deionized water. The solution was filtered through a 0.22 *μ*m filter membrane and 5 *μ*L of resulting solution was injected into the UPLC system for UPLC-MS/MS analysis. For analysis of Zhi-Qiao (7.40 mg) and QZ extracts (45.53 mg), an amount of Zhi-Qiao or QZ extract representing the same amount of raw herb equivalents in the 50 mg of CSGS extract was prepared and analyzed identically to CSGS.

Chromatographic separation was performed on an Acquity UPLC BEH C18 column (2.1 × 100 mm, 1.7 *μ*m, Waters Corp., Milford, USA) using an ACQUITYTM UPLC system (Waters), equipped with a binary solvent delivery system, an autosampler with 4°C, and a PDA detector. The column was maintained at 40°C and eluted at a flow rate of 0.45 mL/min, using a mobile phase of (A) 0.1% (by volume) formic acid in water and (B) acetonitrile. The gradient program was optimized as follows: 0–2 min, 5% B to 10% B; 2–8 min, 10% B to 20% B; 8–10 min, 20% B to 40% B; 10–13 min, 40% B to 90% B; 13–15 min and 90% B to 99% B; 16–20 min, equilibration with 5% B. The mass spectrometric data was collected using Q-TOF analyzer in SYNAPT HDMS system (Waters Corporation, Milford, MA, USA) in both positive and negative ion modes. The source temperature was set at 120°C with a cone gas flow of 30 L/h, a desolvation gas temperature of 450°C with a desolation gas flow of 800 L/h. The capillary voltage was set to 3.0 kV and 2.5 kV for positive and negative ion modes, respectively, and the cone voltage was set to 35 V. Centroid data was collected from  *m*/*z*  50 to 1200 with a scan time of 0.3 s and interscan delay of 0.02 s over a 15 min analysis time. Leucine-enkephalin was used as the lock mass (*m*/*z*  556.2771 in positive mode and  *m*/*z*  554.2615 in negative mode) at a concentration of 0.5 *μ*g/mL with a flow rate of 80 *μ*L/min. The lock spray frequency was set at 20 s.

### 2.7. Monoamine Oxidase A (MAO-A) Activity Assay

MAO was purified from the rat liver according to the Gómez method [13] after minor modification. The liver tissue was rapidly removed into ice cold homogenized (1 : 10, w/v) in 50 mM KH_2_PO_4_ buffer, pH 7.2, containing 0.25 mM sucrose. Following centrifugation at 1000 g for 10 min, the supernatant was centrifuged at 10,000 g for 30 min to obtain crude mitochondrial pellet. 

The MAO-A activity was measured by spectrophotometrically (Mapada, UV-3100, China) using enzymatic kits according to the instructions (Nanjing Jiancheng Institute of Biotechnology). IC_50_ values were used to estimate the inhibition calculated in relation to a sample of the enzyme treated under the same conditions without inhibitor, versus inhibitor concentration.

### 2.8. Computer-Aided Molecular Docking Experiments

Molecular docking of naringenin into the active sites of MAO-A was carried out by using the Windows based software package—Molecular Operating Environment (MOE, Version 2008.10, Chemical Computing Group, Canada). The ligands to be docked were constructed in Discovery Studio (DS, Version 2.5.5, Accelrys Software Inc. USA) and the hydrogen atoms were added according to the appropriate protonation states. Since flexible ligand docking was employed, its geometries required only brief optimization using a fast Dreiding-like force field (1000 iterations) in DS. During the process of optimization, the element, bond orders, number of bonds, and valence were taken into consideration when the terms of the energy equation were calculated. After geometry optimization, the final conformers of ligands were used as starting point for docking. The X-ray crystallographic structures of MAO-A in complex with harmine (PDB code: 2Z5X) were obtained from the Brookhaven Protein Data Bank (http://www.rcsb.org/pdb). Hydrogen atoms were added to the receptor models according to the appropriate protonation states of the ionizable amino acids at pH 7.4 and the valences of the FAD cofactors (oxidized state) and cocrystallized ligands were corrected and hydrogen atoms were added, also according to the appropriate protonation state at pH 7.4. Automated docking was subsequently carried out with the Docking Suit of MOE 2008. Total ligand flexibility was used in this protocol whereby the final ligand conformations were determined by the Monte Carlo conformation search method set to a variable number of trial runs. The docked ligands were further refined using in situ-ligand minimization with the Smart Minimizer algorithm. All parameters for the docking runs were set to their default values and ten possible binding solutions were computed for each docked ligand. The best-ranked binding conformation of ligand was determined according to the Dock Score values. The illustrations were prepared with PyMOL [14].

### 2.9. Data Analysis

To diminish the deviation in data analysis from individual variance of urine samples, the data were normalized by a creatinine calibration method; that is, the metabolite intensity was divided by the creatinine concentration of each sample. Then according to the 80% rule [15, 16], only variables having more than 80% nonzero measurement values were kept in the peak list. Multivariate statistical analysis (MVA) and modeling were performed using Simca-p software (v12.0, Umetric, Umeå, Sweden). Import data were mean-centered and pareto-scaled prior to multivariate analysis. Principal components analysis (PCA) and orthogonal partial least squares discriminate analysis (OPLS-DA) were employed to process the acquired NMR and MS data. PCA was performed to discern the natural separation between different stages of samples by visual inspection of score plots. In the OPLS-DA model, samples from different groups were classified, and the results were visualized in the form of score plots to show the group clusters and S-plots to show the variables contribuing to the classification.

A two-tailed Student's *t*-test was performed using the Statistical Package for Social Science program (SPSS 16.0, SPSS, Chicago, IL, USA). The significance threshold was set at *P* < 0.05 for this test.

## 3. Results

### 3.1. The Antidepressant Effects of CSGS and QZ Extracts on CUMS Treated Rats

The antidepressant effects of CSGS and QZ extracts were evaluated on a CUMS rat model, a well-validated animal model of depression (see supplementary material Table S1 available online at http://dx.doi.org/10.1155/2013/487158). The body weight, the number of horizontal movements, and the sucrose preference [17, 18] in the CUMS treated rats decreased significantly compared with control group. Rats treated with CSGS extract showed significant increases in the body weight, the number of horizontal movements, and the sucrose preference compared with CUMS group. Similar changes were observed in the QZ treated group except the number of horizontal movements in rats. The results indicated that QZ treated had similar therapeutic effect to CSGS in increasing body weight and sucrose preference, but did not improve the rat horizontal movement significantly compared with CSGS treated. 

### 3.2. Urinary Metabonomics Study by ^1^H NMR and UPLC-Q-TOF/MS

#### 3.2.1. Metabolic Profiles of CUMS-Induced Depression with CSGS and QZ Treatments

**Figure 2:**
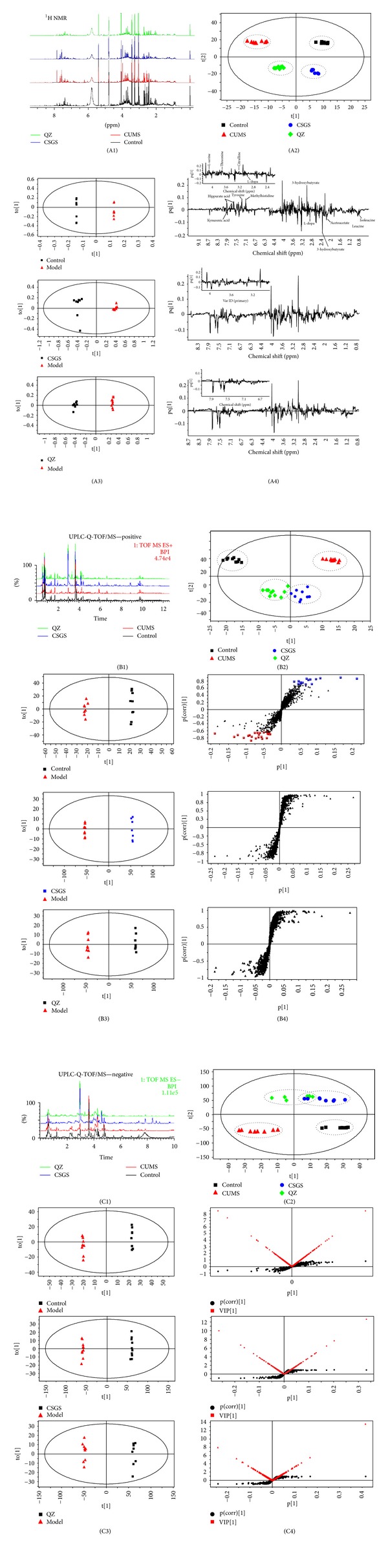
MVA of urine samples from all groups detected by NMR and UPLC-Q-TOF/MS. A1, B1, and C1: the spectra and chromatograms of urine samples. Black color stands for control group, red color for CUMS group, blue color for CSGS treated group, and green color for QZ treated group; A2, B2, and C2. The score plots of control, CUMS, CSGS, and QZ groups; A3, B3, and C3. OPLS-DA scores plots of CUMS versus model groups, CUMS versus CSGS groups, CUMS versus QZ groups (A3: *Q*2*X*_CUM_ = 0.879, *R* ^2^ *Y*_CUM_ = 0.998, *Q*_(CUM)_^2^ = 0.607, B3: *Q* ^2^ *X*_(CUM)_ = 0.604, *R* ^2^ *Y*_CUM_ = 0.994, *Q*_(CUM)_^2^ = 0.99; C3: *Q* ^2^ *X*_(CUM)_ = 0.659, *R* ^2^ *Y*_CUM_ = 0.999, *Q*_(CUM)_^2^ = 0.999); A4, B4, and C4: line plots, S-plots, and VIP-value plots of urine samples detected by NMR and UPLC-Q-TOF/MS, respectively.

The urine samples collected on the 28th day from each group were analyzed by ^1^H NMR (Figure S1 in supplementary material) and UPLC-Q-TOF/MS (Figures 2(A1), 2(B1), and 2(C1)). Principal components analysis (PCA) (Figures 2(A2), 2(B2), and 2(C2)) indicated that the metabolic profile of rat in CUMS model group deviated from the control, suggesting that significant biochemical changes were induced by CUMS. The metabolic profile of rats in CSGS treated group fairly differed from the CUMS group and was close to the control, indicating that the deviations induced by CUMS were significantly improved after treatment of CSGS. Similar results were observed in QZ treated group except that the mean center of spots in CSGS treated group was much closer to control group than that of QZ treated group, which was consistent with the results of ethological study. 

#### 3.2.2. Potential Biomarkers in the CUMS-Induced Depression Associated with Treatment of CSGS or QZ

OPLS-DA is a supervised multivariable statistical method to sharpen an already established separation between each two groups in PCA. In order to obtain better discrimination between CUMS and treatment groups, OPLS-DA was performed. As shown in Figures 2(A3), 2(B3), and 2(C3), the urine metabolic profiles of the control and CUMS groups, of the CSGS treated and CUMS groups, and of the QZ treated and CUMS groups were clearly separated. 

**Figure 3:**
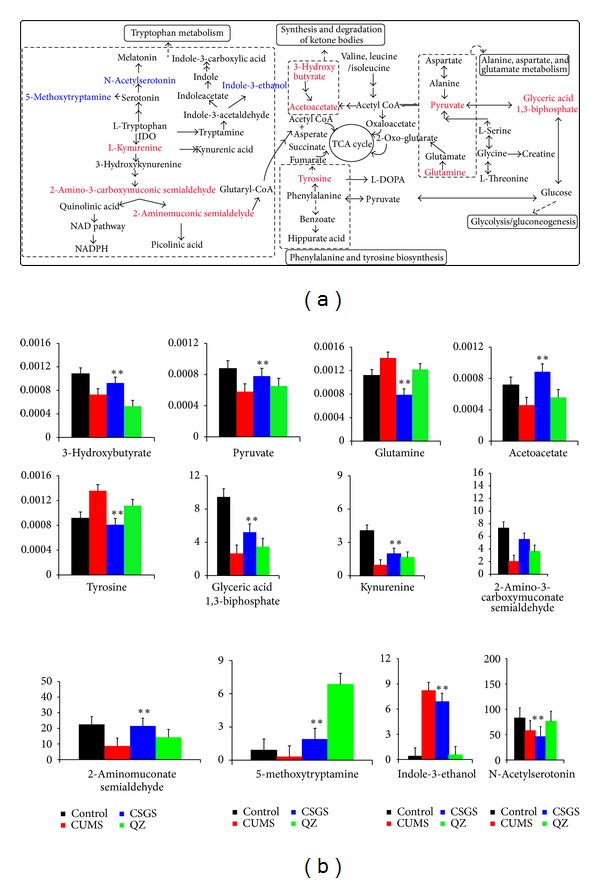
(a) Urinary metabolic pathways of CUMS-induced depression with CSGS or QZ treatment. Metabolites in red font: the potential biomarkers on which CSGS had regulations but QZ did not; metabolites in blue font: the potential biomarkers on which QZ had regulations but CSGS did not. (b) The levels of 12 potential biomarkers for differentiating CSGS and QZ treated. (*P < 0.05 versus CUMS group, **P < 0.01 versus CUMS group).

According to the S-plot, those variables with lager VIP values (VIP > 1) were selected as key metabolites for the differentiation between CUMS group and the control group, and the CSGS treated and CUMS groups, and the QZ treated and CUMS groups. If the metabolites not only represented the difference between CUMS group and the control group, but also the difference between CUMS group and CSGS or QZ treated group, they were considered as potential biomarkers of metabolic deviations in CUMS-induced depression mediated by CSGS or QZ (Figures 2(A4), 2(B4) and 2(C4)). As a result, twenty-eight variables were identified as potential biomarkers (Table 1 and Figure 3(a)). Fourteen of them were detected by ^1^H NMR and the others were detected by UPLC-Q-TOF/MS.

Here, a potential biomarker with  *m*/*z*  154.1628 at 1.07 min was taken as an example to illustrate the identification process. First, full-scan mass spectrum of the peak at 1.07 min (Figure S2A in supplementary material) in the positive ion mode provided a quasimolecular ion  at *m*/*z*  154.1628, suggesting a molecular formula with [C_8_H_11_NO_2_]^+^ (calculated to be 154.1789). Candidates were obtained in searching molecular weight at 154.1628 Da (positive mode, MW tolerance ±0.05 Da) from database including MassBank (http://www.massbank.jp/index.html), HMDB (http://www.hmdb.ca), and METLIN (http://metlin.scripps.edu). As a result, there are two candidates with molecular weight at 154.1628 ± 0.05 Da, which are described as norphenylephrine and dopamine, respectively. The MS^2^ spectrum of the ion at  *m*/*z*  154.1628 (Figure S2B in supplementary material) generated a series of ions at  *m*/*z*  137.1699, 119.1435, 109.1635 and 91.1547, which are in agreement with the fragmentation pattern of dopamine [19] (Figure S3 supplementary material). 

#### 3.2.3. Difference between CSGS and QZ in Correction of Metabolic Deviations Induced by CUMS

The variation tendency of the identified potential biomarkers in CUMS model, CSGS treated, and QZ treated was shown in Table 1. The concentrations of sixteen metabolites (**1**–**4**, **10**–**13**, **19**–**26**) were significantly decreased and twelve increased in CUMS group compared with normal control. Obviously, these metabolites were associated with depression-induced injury. 

The deviations of all potential biomarkers induced by CUMS were corrected with CSGS treated except N-acetylserotonin (**26**), indole-3-ethanol (**27**), and 5-methoxytryptamine (**28**). The concentrations of fifteen metabolites (**1**–**4**, **10**–**13**,** 19**–**25**) were upregulated and the other ten metabolites (**5**–**9**, **14**–**18**) were downregulated by CSGS, which represents the CSGS medication on chronic stress. 

More importantly, QZ did not show any improvement on the deviations of nine variables (**10**–**14**, **22**–**25**) (Figure 3) induced by CUMS, and the mediation effect of QZ on isoleucine (**2**), glutamate (**3**), and L-dopa (**4**) had no statistical significance as well. The results may explain why the metabolic pattern of rats in QZ treated group was not closer to the normal control group than that of CSGS treated group and suggested that the mediation effects of CSGS on the metabolites **2**–**4**, **10**–**14**, and **22**–**25** were lost in QZ. In other words, those mediations of CSGS should be provided by Zhi-Qiao.

### 3.3. Identification of the Chemical Constitutes Responsible for Those Regulations of CSGS Missed in QZ

**Figure 4:**
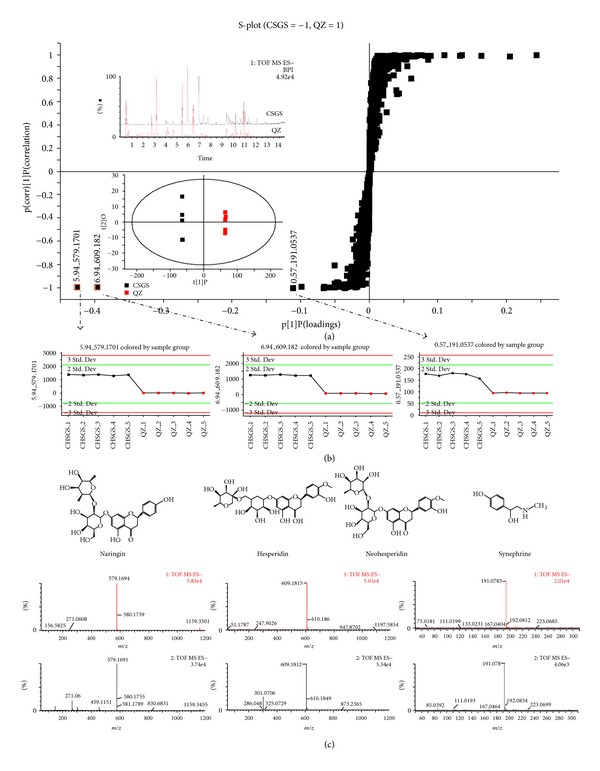
MVA on chemical profiles of the CSGS and QZ extracts. (a) Score plots in OPLS-DA analysis of CSGS and QZ. (b) Trend plots: the level of the variables in the CSGS (black spot) and QZ extracts (red spot). (c) The structures and MS spectra of synephrine, naringin, and hesperidin/neohesperidin.

Further, comparison of the chemical profiles among CSGS, QZ, and Zhi-Qiao was conducted for identification of the chemical constituents responsible for those mediation effects of CSGS lost in QZ, that is, regulations on the metabolites **2**–**4**, **10**–**14**, and **22**–**25**. The extracts of CSGS, QZ (CSGS without Zhi-Qiao), and Zhi-Qiao were analyzed by using UPLC-Q-TOF/MS under the same conditions (Figures S4 and S5). Compared with the chemical profile of CSGS, some of the peaks disappeared and the intensities of some peaks were changed in QZ extracts. OPLS-DA was applied in finding the differences between CSGS and QZ extracts in the chemical profile. The variables responsible for the differentiation between the CSGS and QZ extracts were identified from S-plots; the *Y* ^+^ axis represented the QZ extract; the  *Y* ^−^  axis represented the CSGS extract; the *X*-axis represented the number of detected ions (Figure 4(a)). Three candidate ions with retention time and  *m*/*z*  pairs at 0.57_191.0937 (i), 5.94_579.1701 (ii), and 6.94_609.1820 (iii) located furthest from the origin in the *Y* ^−^ axis, contributing significantly to discriminating CSGS from QZ. In the trend plots (Figure 4(b)), the concentrations of those compounds in QZ extract were nearly zero, which indicated that these compounds came from the subtracted herb, Zhi-Qiao. Based on the retention behaviors, accurate molecular weight and MS/MS fragments from UPLC-Q-TOF/MS analysis on Zhi-Qiao extract (Figure 3(c)), and by comparison with reference standards, were identified as synephrine (i), naringin (ii), and hesperidin (iii**-1**)/neohesperidin (iii**-2**), respectively. They are the chemical contributors to discriminate CSGS from QZ extracts and also are the active constituents of CSGS from Zhi-Qiao to mediate the deviations of the metabolites **2**–**4**, **10**–**14**, and **22**–**25** induced by CUMS.

### 3.4. Active Evaluation of the Active Constituents from Zhi-Qiao against Monoamine Oxidase A (MAO-A)

Monoamine oxidase A (MAO-A), an enzyme presenting in the outer mitochondrial membrane of neuronal and nonneuronal cells, catalyses the oxidative domination of primary, secondary, and tertiary amines [20]. The regulation of MAO-A activity appeared to play a central role in several psychiatric and neurological disorders. Thus, inhibition of MAO-A may alleviate symptoms of depression [21].

The inhibition of synephrine, naringin, hesperidin, and neohesperidin against MAO-A activity was assayed by employing rat MAO-A as enzyme source and moclobemide as positive control. Naringin showed the highest activity with an IC_50_ value of 5.82 *μ*M (Table 2). The IC_50_ values of synephrine, hesperidin, and neohesperidin were 15.23 *μ*M, 26.72 *μ*M, and 93.76 *μ*M.

### 3.5. Molecular Docking Studies

Molecular docking with the MOE 2008 was carried out with the aim of understanding the possible binding orientations of naringin within the MAO active sites. The crystallographic structures of human MAO-A cocrystallized with harmine (PDB entry: 2Z5X) served as protein model. The protein model and docking protocol have previously been shown to be appropriate for predicting binding orientations of ligands in the active sites of MAO-A. Primary experiment has indicated that naringin was poorly absorbed from the gastrointestinal tract in its original form and must be hydrolyzed by microflora enzymes (bacterial *β*-glucuronidase) in gut to its aglycone (naringenin) in human and rat [22, 23]. Thus, naringenin was subjected molecular docking with the MOE 2008. We found that the aglycone of naringin was able to form a relatively stable docking structure with MAO-A through our docking experiment. 

**Figure 5:**
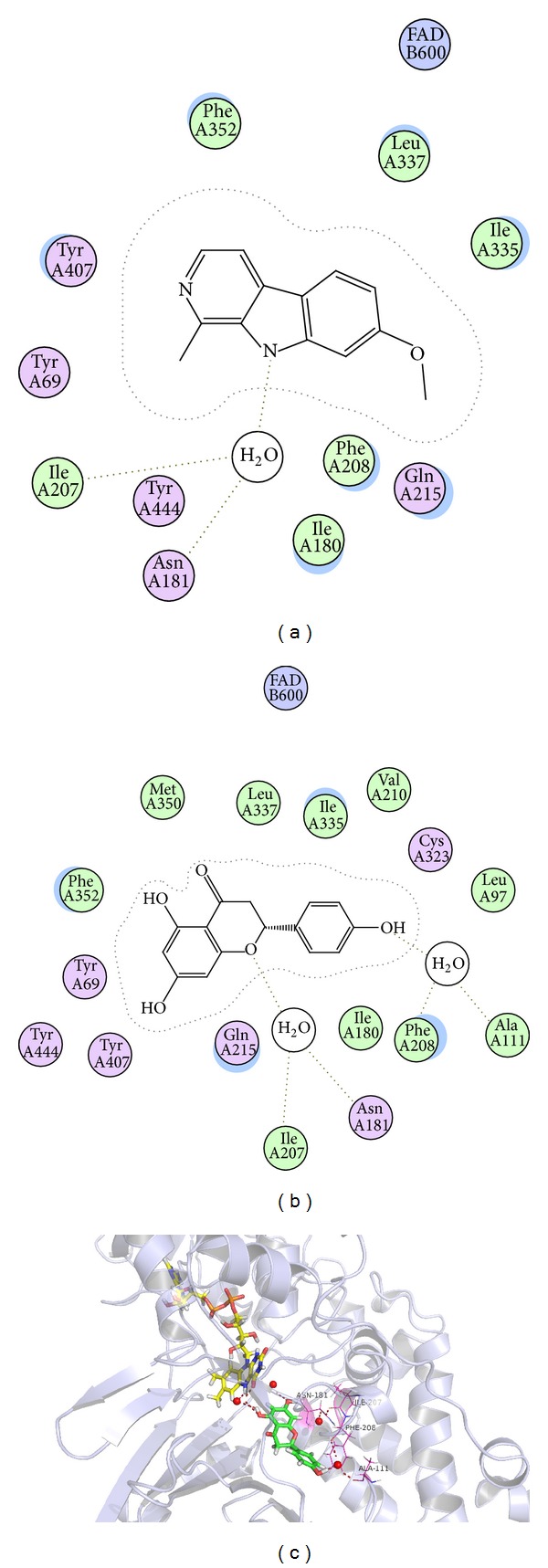
Computer-aided molecular docking. (a) The 2D map of predicted binding orientation of harmine within the MAO-A active site. (b) The 2D map of predicted binding orientation of naringenin within the MAO-A active site. (c) The 3D map of predicted binding mode of naringenin within the active site of MAO-A.

The highest ranked solution of harmine (positive control) and naringenin within the MAO-A active site was illustrated in Figures 5(a) and 5(b). The results showed that both naringenin and harmine bond within the substrate cavity of the enzyme through heteroatom of the heterocyclic and water molecule as a bridge which linked activity compounds and enzyme. As shown in Figures 5(b) and 5(c), the benzene ring of the naringenin binds within the substrate cavity of the enzyme and undergoes potential hydrogen bonding with FAD cofactor by a water molecule. In addition, the oxygen atom of pynan ring in naringenin undergoes potential hydrogen bonding with the phenolic hydrogen of Ile-207, Asn-181 which was shown in Figure 5(b). Furthermore, the phenolic hydroxyl of benzene ring of naringenin undergoes potential hydrogen bonding with the phenolic hydrogen of Phe-208, Ala-111. The experimental results showed that naringenin is able to connect steadily with MAO-A through water molecule and could be viewed as a promising compound for the inhibition of MAO-A activity.

## 4. Discussion

Traditional Chinese herbal medicine typically uses mixtures of many herbs to treat a given disease. It is believed that complex interactions produce synergistic effects and reduce possible side effects from some herbs. The conventional strategy applied in screening bioactive components in TCMs is isolation and identification of the individual components one by one and then evaluation of their activities *in vitro*/*in vivo*. This strategy is not only time consuming and labor intensive, but also often fails to discover the bioactive constituents contributing to the efficacy of TCM formulas. A very important reason is that no synergistic effects among diverse chemical constituents in herbal medicines are considered.

Chaihu-Shu-Gan-San (CSGS) has been proved to be an effective TCMs formula for treatment of depression and is featured as multiingredients preparation and multitargets intervention on the systemic level [11]. However, the chemical constituents of CSGS related to its efficacy were still not clear, even though some constituents with antidepressant effect were reported from the single herbs of CSGS, such as saikosides (Chai-Hu) [24], total glycosides of paeonia (TGP, Bai-Shao) [25], and liquiritin (Gan-Cao) [26].

Metabonomics is focused on measuring the overall metabolites of biological samples which coincides with the holistic thinking of TCM. It enables the parallel assessment of the levels of a broad range of endogenous and exogenous metabolites [27] and has shown potential in evaluation of therapeutic effect of TCMs [9].

In our research, metabonomics provided a visualized pattern and relative quantitative estimate to help us acknowledge the potential biomarkers of CUMS induced depression and the different regulations after CSGS and QZ treatments. The results-indicated that CSGS showed more comprehensive regulations than QZ after 28 days of therapeutic intervention. QZ did not show effect on twelve potential biomarkers (**2**–**4**, **10**–**14**, **22**–**25**) as CSGS did. The different regulations between CSGS and QZ treated groups suggested that the subtracted herb, that is, Zhi-Qiao, was responsible for the missing regulations of QZ compared to CSGS. The findings indicated that Zhi-Qiao contributed to the antidepressant effect of CSGS through regulating the deviations of the potential biomarkers **2**–**4**, **10**–**14**, **22**–**25** (Table 1 and Figure 3). Among them, isoleucine (**2**), glutamate (**3**), dopa (**4**), acetoacetate (**10**), 3-hydroxybutyrate (**11**), pyruvate** (12**), glutamine (**13**), tyrosine (**14**), and glyceric acid 1, 3-biphosphate (**22**) were involved in metabolic pathways associated with the onset of CUMS-induced depression in our previous study [10], including ketone bodies synthesis, phenylalanine, tyrosine and tryptophan biosynthesis, valine, aspartate and glutamate metabolism, and glycolysis/gluconeogenesis. The other three potential biomarkers, kynurenine (**23**), 2-aminomuconate semialdehyde (**24**), and 2-amino-3-carboxymuconate semialdehyde (**25**), are the main products in kynurenine pathway (a major route of tryptophan metabolism). Tryptophan and its metabolites such as serotonin and kynurenines (KYNs) are strong modulators of emotional behavior in the central nervous system (CNS) [28] and have been implicated in brain dysfunction in several disorders such as Huntington's disease, Alzheimer's disease, and depression [28–30]. Obviously, Zhi-Qiao participated in the regulation of tryptophan metabolism, ketone bodies synthesis, phenylalanine, tyrosine and tryptophan biosynthesis, valine, aspartate, glutamate metabolism and glycolysis/gluconeogenesis to assist CSGS treated of depression.

Interestingly, QZ showed regulations on three of the potential biomarkers, N-acetylserotonin (**26**), indole-3-ethanol (**27**), and 5-methoxytryptamine (**28**) (Figure 3(b)), but CSGS did not have effects on these three metabolites. They are the products of 5-HT synthesis, one of the pathways in tryptophan metabolism. The results suggested that QZ influenced the synthesis of 5-HT because there exist active constituents in QZ (i.e., all single herbs of CSGS except Zhi-Qiao) and competing constituents in Zhi-Qiao. CSGS did not have effect on the synthesis of 5-HT; however, it could mediate the disturbances of tryptophan metabolism induced by CUMS through kynurenine pathways (KYP), the other pathways in tryptophan metabolism.

Undoubtedly, the mediations of CSGS missed in QZ should come from Zhi-Qiao, the single herb subtracted from CSGS. However, not all constituents in Zhi-Qiao make contributions to those missing medications. In order to know which compounds from Zhi-Qiao are associated with those missing medications, MVA was performed on the chemical profiles of CSGS and QZ extracts. Four variables shown in the S-plots of OPLS-DA were recognized as significant contributors to discriminate the chemical profiles of CSGS and QZ extracts. They represented the antidepressant ingredients of CSGS from Zhi-Qiao, and were identified as synephrine, naringin, hesperidin, and neohesperidin, respectively. 

To the best of our knowledge, this is the first report for the antidepressant activity naringin, hesperidin, and neohesperidin. Antidepressant-like effect of synephrine was tested in mouse models of immobility tests [31]. The *in vitro* MAO-A inhibitory assay confirmed their antidepressant effect using rat MAO-A as enzyme source and moclobemide as positive control. Naringin showed the highest activity with an IC_50_ value of 5.82 *μ*M, while synephrine, hesperidin, and neohesperidin were found to be relatively weak. The possible binding orientation of naringin within the MAO active sites was speculated by molecular docking study and the results suggested that the aglycone of naringin and naringenin [21, 22] was fused with MAO-A by a hydrogen bond through water molecule. 

## 5. Conclusions

This paper integrated metabonomics and chemical profile in combination with the multivariate statistical analysis (MVA) to discover the chemical constituents which contribute to the efficacy of CSGS. Through the difference between CSGS and QZ extracts in chemical profiles and the regulations of metabolic perturbations induced by CUMS, four compounds were identified as the active constituents of CSGS contributed by Zhi-Qiao. They were responsible for those regulations of CSGS on the deviations of the metabolites **2**–**4**, **10**–**14**, and **22**–**25**, involved in metabolic dysfunction of ketone bodies synthesis, phenylalanine, tyrosine and tryptophan biosynthesis, valine, aspartate, glutamate metabolism, and glycolysis/gluconeogenesis. The potential antidepressant activities of the four compounds were evaluated by MAO-A activity assay *in vitro*. Naringin showed the potent inhibition against MAO-A activity with an IC_50_ value of 5.82 *μ*M. 

Accordingly, we ascertained the contributions of Zhi-Qiao to the CSGS antidepressant effect. In future, the integration of metabonomics and chemical profile could be used to clarify the contributions of all single herbs in CSGS and uncover all active constituents of CSGS. Our findings provide a new effective strategy for uncovering what the active chemical constituents in TCM formula are and how they make contributions for the efficacy of the formula.

## Supplementary Material

The supporting information depicted the antidepressant effects of CSGS and QZ extracts on CUMS treated rats. Meanwhile, the typical ^1^H NMR spectrum of urine sample; the BPI chromatograms of the extracts of CSGS, QZ and Zhi-Qiao; the spectra and fragmentation pathway of dopamine were also provided.

## Figures and Tables

**Table 1:** The potential biomarkers of CUMS-induced depression and their variation tendency after oral administration of CSGS or QZ extract for 28 days.

NMR						UPLC-Q-TOF/MS									
No.	Metabolites	*δ* (ppm) and multiplicity	Trend			No.	Metabolites	TR	*M*/*Z*	Selected ion	Elemental composition	Fragment of MS/MS	Trend		
			CUMS^c^	CSGS^d^	QZ^d^								CUMS^c^	CSGS^d^	QZ^d^
1	Lipid(CH_3_CH_2_)	0.94 (t)	↓**	↑**	↑**	15	Dopamine^a^	1.07	154.1628	[M+H]^+^	C_8_H_11_NO_2_	154.1628; 119.1435; 109.1635; 91.1547	↑**	↓**	↓**
2	Isoleucine	0.94 (t)	↓**	↑**	↑	16	Histamine^b^	0.7	112.0727	[M−H]^−^	C_5_H_9_N_3_	112.0727; 95.0653	↑**	↓**	↓**
3	Glutamate	2.36 (m)	↓**	↑**	↑	17	Isobutyrylglycine^b^	1.15	144.1583	[M−H]^−^	C_6_H_11_NO_3_	128.2656; 74.1324	↑**	↓**	↓**
4	L-dopa	2.96 (d)	↓**	↑**	↑	18	2-phenylethanol glucuronide^b^	5.94	297.0976	[M−H]^−^	C_14_H_18_O_7_	ND	↑**	↓**	↓**
5	**α**-glucose	3.86 (dd), 5.23 (d)	↑**	↓**	↓**	19	3-hydroxyhippuric acid^a^	4.36	195.1846	[M+H]^+^	C_9_H_9_NO_4_	195.1846; 180.0856	↓**	↑**	↑**
6	L-serine	3.96 (d)	↑**	↓**	↓**	20	Creatine^a^	4.36	132.0951	[M+H]^+^	C_4_H_9_N_3_O_2_	132.0951; 90.1743	↓**	↑**	↑**
7	Phenylalanine	3.97, 7.32 (m)	↑**	↓**	↓**	21	5-aminoimidazole-4-carboxamide^b^	2.93	125.0874	[M−H]^−^	C_4_H_6_N_4_O	ND	↓**	↑**	↑**
8	Hippurate acid	7.55 (m), 7.57 (m), 7.84 (d)	↑**	↓**	↓**	22	Glyceric acid 1,3-biphosphate^a^	0.59	267.0585	[M+H]^+^	C_3_H_8_O_10_P_2_	267.0585; 250.0323	↓**	↑**	—
9	Kynurenic acid	7.64 (d)	↑**	↓**	↓**	23	L-kynurenine^a^	0.64	209.0795	[M+H]^+^	C_10_H_12_N_2_O_3_	209.0795; 192.1320; 94.3122	↓**	↑**	—
10	Acetoacetate	2.22 (s)	↓**	↑**	—	24	2-aminomuconate semialdehyde^b^	9.29	140.1169	[M−H]^−^	C_6_H_7_NO_3_	ND	↓**	↑**	—
11	3-hydroxybutyrate	2.31 (d), 2.33 (m), 2.38 (m)	↓**	↑**	—	25	2-amino-3-carboxymuconate semialdehyde^b^	9.69	184.1278	[M−H]^−^	C_7_H_7_NO_5_	ND	↓**	↑**	—
12	Pyruvate	2.36 (s)	↓**	↑**	—	26	N-acetylserotonin^b^	4.68	217.1081	[M−H]^−^	C_12_H_14_N_2_O_2_	ND	↓**	—	↑**
13	Glutamine	2.08 (m)	↓**	↑**	—	27	Indole-3-ethanol^a^	3.37	162.1183	[M+H]^+^	C_10_H_11_NO	162.1183; 144.1054	↑**	—	↓**
14	Tyrosine	7.22 (m)	↑**	↓**	—	28	5-methoxytryptamine^a^	1.26	191.0824	[M+H]^+^	C_11_H_14_N_2_O	191.0824; 173.0807; 160.097	↑**	—	↓**

^a^The potential biomarkers were detected by UPLC-Q-TOF/MS in positive mode.

^b^The potential biomarkers were detected by UPLC-Q-TOF/MS in negative mode.

^c^Chang trend compared to control group.

^d^Chang trend compared to CUMS group.

The levels of potential biomarkers were labeled with (↓) downregulated and (↑) upregulated (*P < 0.05; **P < 0.01); (—) represents no statistically significant difference.

CUMS: model group; CSGS: CSGS treated group with CUMS; QZ: QZ treated group with CUMS.

**Table 2:** Inhibition of synephrine, naringin, hesperidin, and neohesperidin against MAO-A activity.

No.	Compounds	IC_50_ (*μ*M)
i	Synephrine	15.23
ii	Naringin	5.82
iii-**1**	Hesperidin	26.72
iii-**2**	Neohesperidin	93.76
Control	Moclobemide	5.25

## Abbreviations

CSGS: Chaihu-Shu-Gan-San
MVA: Multivariate statistical analysis
Zhi-Qiao: *Citrus aurantium* L.
QZ: Chaihu-Shu-Gan-San without Zhi-Qiao
CUMS: Chronic unpredicted mild stress
MAO-A: Monoamine oxidase A
TCMs: Traditional Chinese medicines
QC: Quality control
TSP: Sodium-3-trimethylsilyl-[2, 2, 3, 3-^2^H_4_]-1-propionate
PCA: Principal components analysis
OPLS-DA: Orthogonal to partial least squares-discriminate analysis
CNS: Central nervous system
RT: Retention time.
