# Supplementary material for: Metabonomics Combined with UPLC-MS Chemical Profile for Discovery of Antidepressant Ingredients of a Traditional Chinese Medicines Formula, Chaihu-Shu-Gan-San
Source: Evid Based Complement Alternat Med. 2013 Apr 4;2013:487158. doi: 10.1155/2013/487158 (PMC3638638; doi:10.1155/2013/487158)
Supplement: Supplementary file 1 — The supporting information depicted the antidepressant effects of CSGS and QZ extracts on CUMS treated rats. Meanwhile, the typical 1H NMR spectrum of urine sample; the BPI chromatograms of the extracts of CSGS, QZ and Zhi-Qiao; the spectra and fragmentation pathway of dopamine were also provided. [file 487158.f1.pdf]

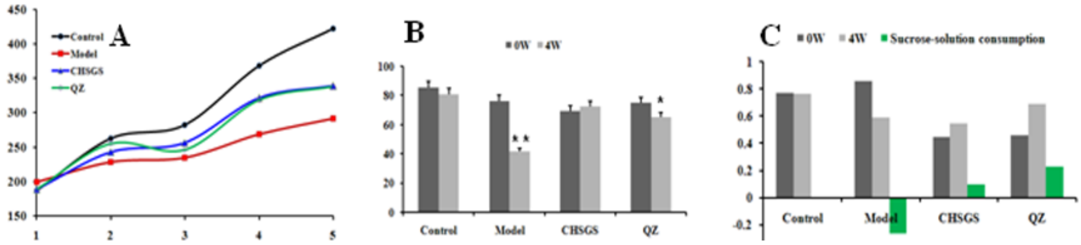

Figure S1: Ethological study of the anti-depressive effect of CSGS and QZ extracts on CUMS treated rats. (A) Body weight changes. The weight growth rate of CUMS (red color) was significantly lower than the control group (black color). The weight gain of CSGS and QZ treatment groups are increased compared to CUMS, but there is no significant difference between the two groups. (B) Open-field test. The horizontal movement is decreased significantly after CUMS treated. Both of CSGS and QZ treatments improved the horizontal movement of CUMS rats. (C) Sucrose preference test. The sucrose solution consumption (green color) of control group showed no alternated after 4 weeks. The sucrose solution consumption of CUMS treated rats decreased significantly after 4 week treatment. CSGS and QZ treatment groups can improve the sucrose solution consumption after continuous drug treatment.

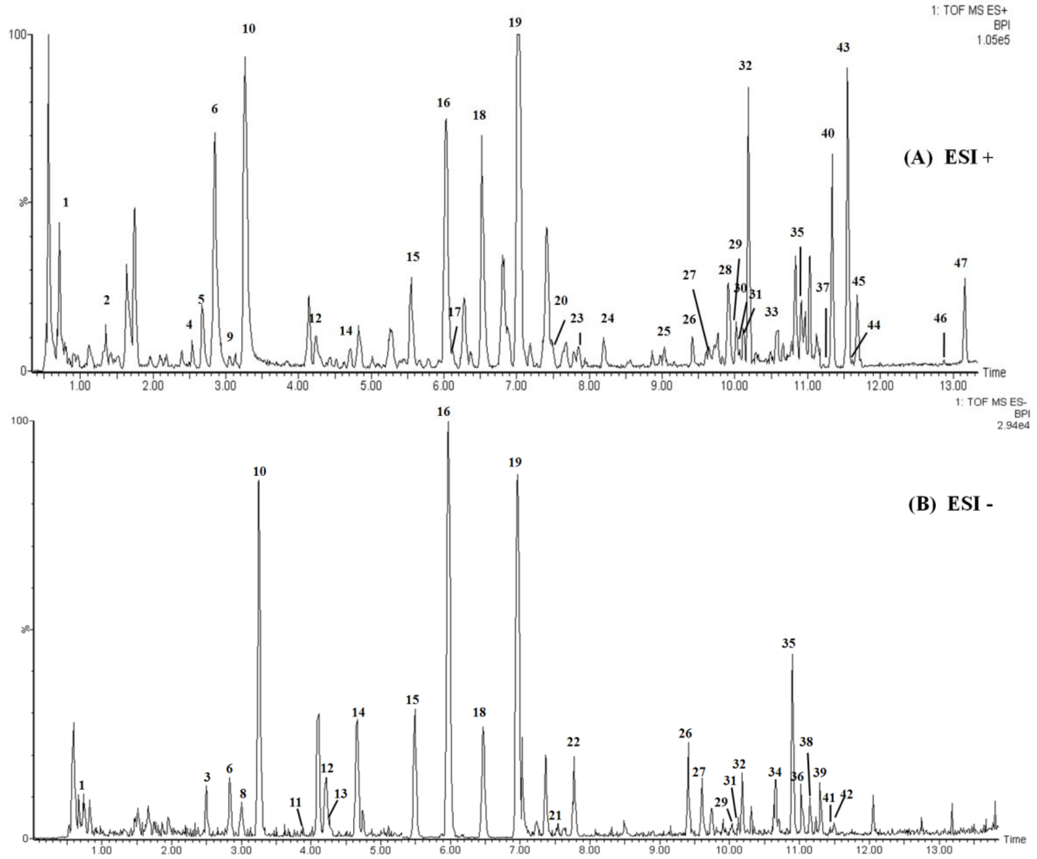

Figure S2: The fingerprint profiles of CSGS samples by UPLC-Q-TOF/MS. (A) Positive ESI mode. (B) Negative ESI mode. Peaks 4, 28, 39, 41 and 42 originated from *Bupleurum chinense* DC. (Chai-Hu); Peaks 1, 18, 26, 27, 31, 40, 43 and 45 originated from *Citrus reticulata* Blanco (Chen-Pi), Peaks 3, 4, 6, 7, 10, 29 and 32 originated from *Paeonia lactiflora* Pall. (Bai-Shao), Peaks 14-19, 24, 26, 27, 30, 31 and 45 from *Citrus aurantium* L. (Zhi-Qiao), Peaks 2, 25, 28 and 37 originated from *Cyperus rotundus* L. (Xiang-Fu), Peaks 5, 11, 20, 44 and 46 originated from *Ligusticum chuanxiong* Hort. (Chuan-Xiong) and Peaks 8, 12, 13, 21-23, 33, 35, 36 and 38 originated from *Glycyrrhiza uralensis* Fisch. (Gan-Cao).

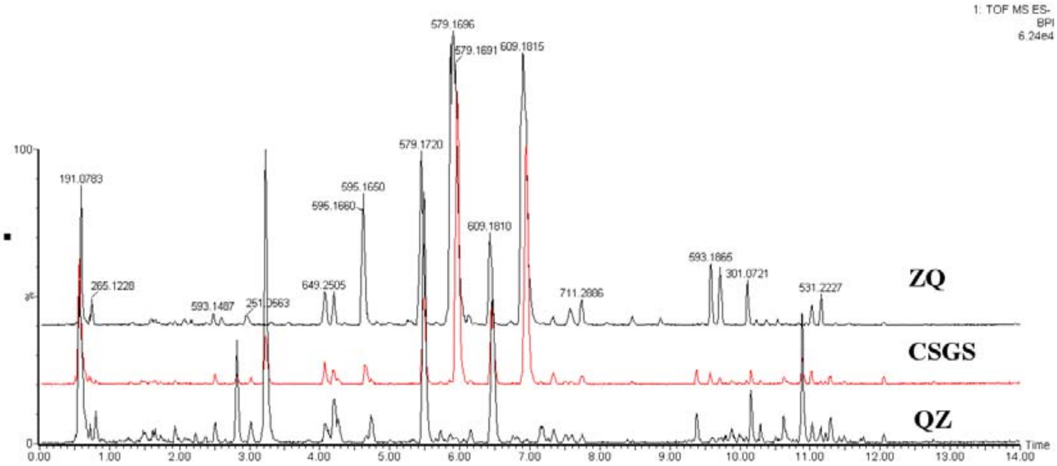

Figure S3: The BPI chromatography of the extracts of CSGS, QZ and Zhi-Qiao in negative mode.
