# Supplementary material for: Metabonomics Combined with UPLC-MS Chemical Profile for Discovery of Antidepressant Ingredients of a Traditional Chinese Medicines Formula, Chaihu-Shu-Gan-San
Source: Evid Based Complement Alternat Med. 2013 Apr 4;2013:487158. doi: 10.1155/2013/487158 (PMC3638638; doi:10.1155/2013/487158)
Supplement: Supplementary file 2 [file 487158.f2.pdf]

Table S1. The antidepressant effects of CSGS and QZ extracts on CUMS treated rats ($\bar{x}$ ±S.E.M, n = 8)

Group	Body weight（g）		Open-field test (OFT)		Sucrose consumption（g）	
	0W	4W	0W	4W	0W	4W
Control	186.78±10.57	235.65±12.37	85.6±9.82	81.1±8.43	17.2	17.1
CUMS[a]	199.16±16.34	93.12±6.89**	76.5±7.92	42.0±8.31**	16.7	8.4**
Positive control[b]	191.702±15.02	144.342±17.21*	81.7±9.84	61.4±7.82*	15.8	17.5*
CSGS[b]	189.08±11.46	150.522±14.14*	69.6±7.43	72.43±9.82	16.5	17.3
QZ[b]	188.71±13.71	147.16±14.48*	75.2±6.73	65.4±5.53	17.6	21.7**

[a] ** $p < 0.01$ vs control group, *$p < 0.05$ vs control group

[b] ** $p < 0.01$ vs CUMS group, *$p < 0.05$ vs CUMS group

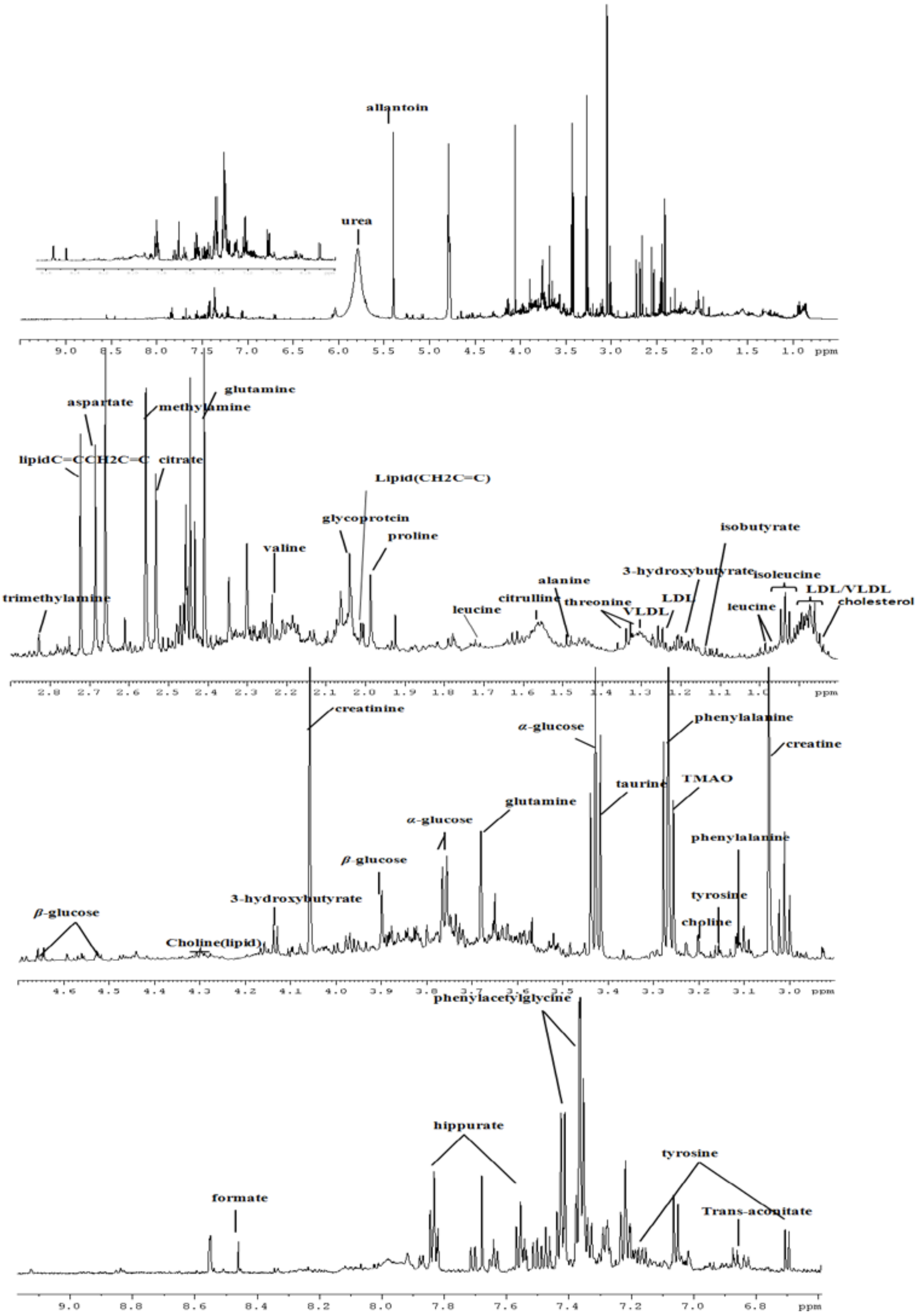

Figure S1. Typical ^{1}H NMR spectrum of a urine sample of rats in control group.

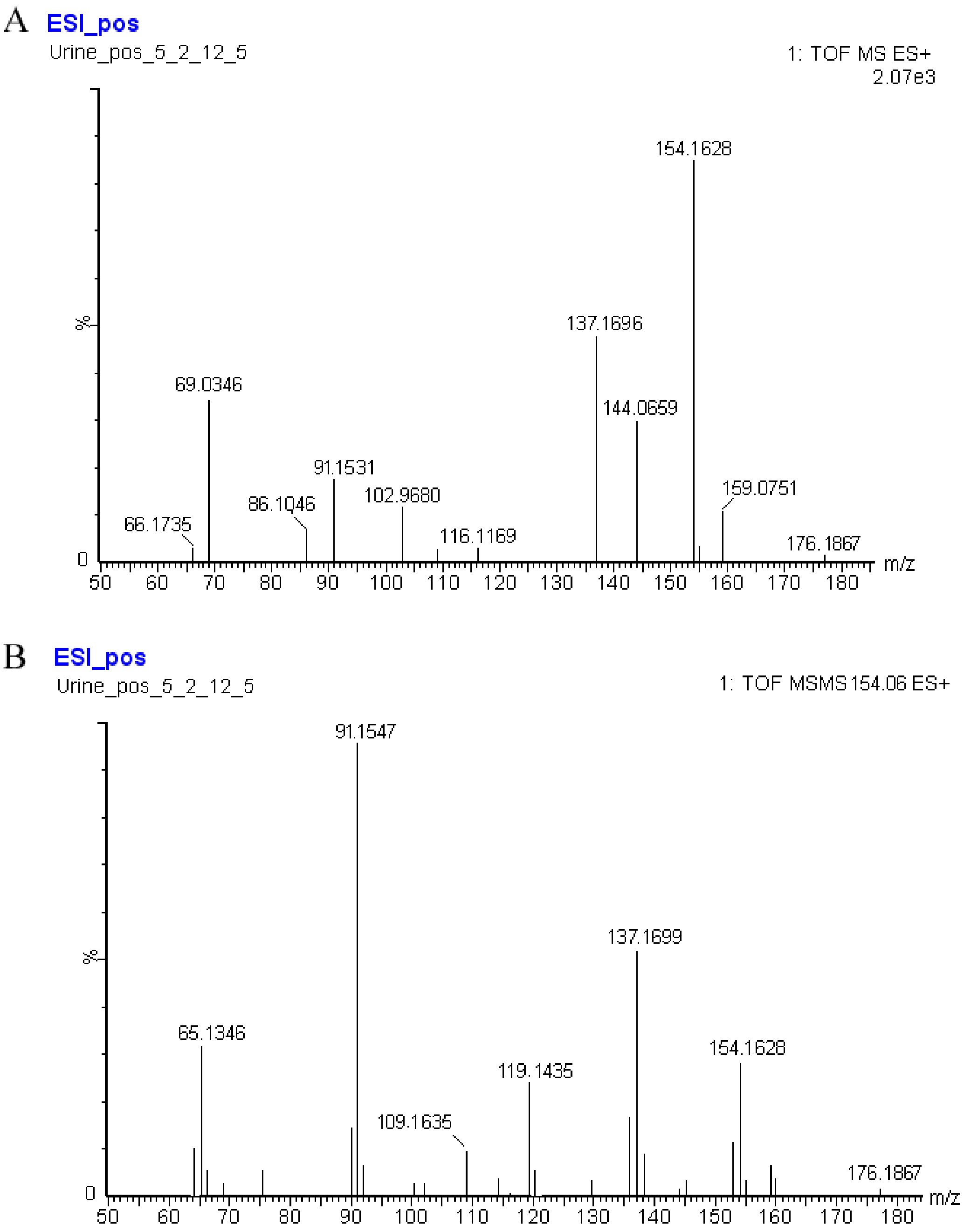

Figure S2: (A) Full-scan mass spectrum of the peak *m/z* 154.1628 at 1.07 minute in positive ion mode, (B) MS^2 spectrum of ion at *m/z* 154.1628 in positive ion mode.

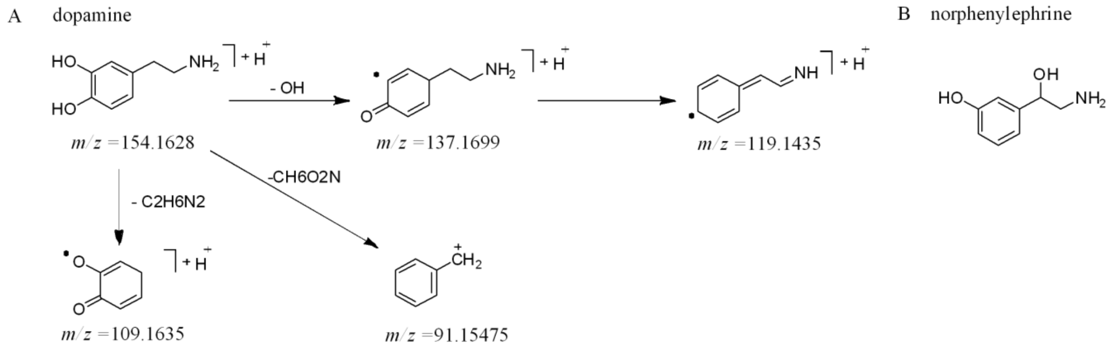

Figure S3: A, Fragmentation pathway of dopamine in positive ion mode. B. The structure of norphenylephrine.

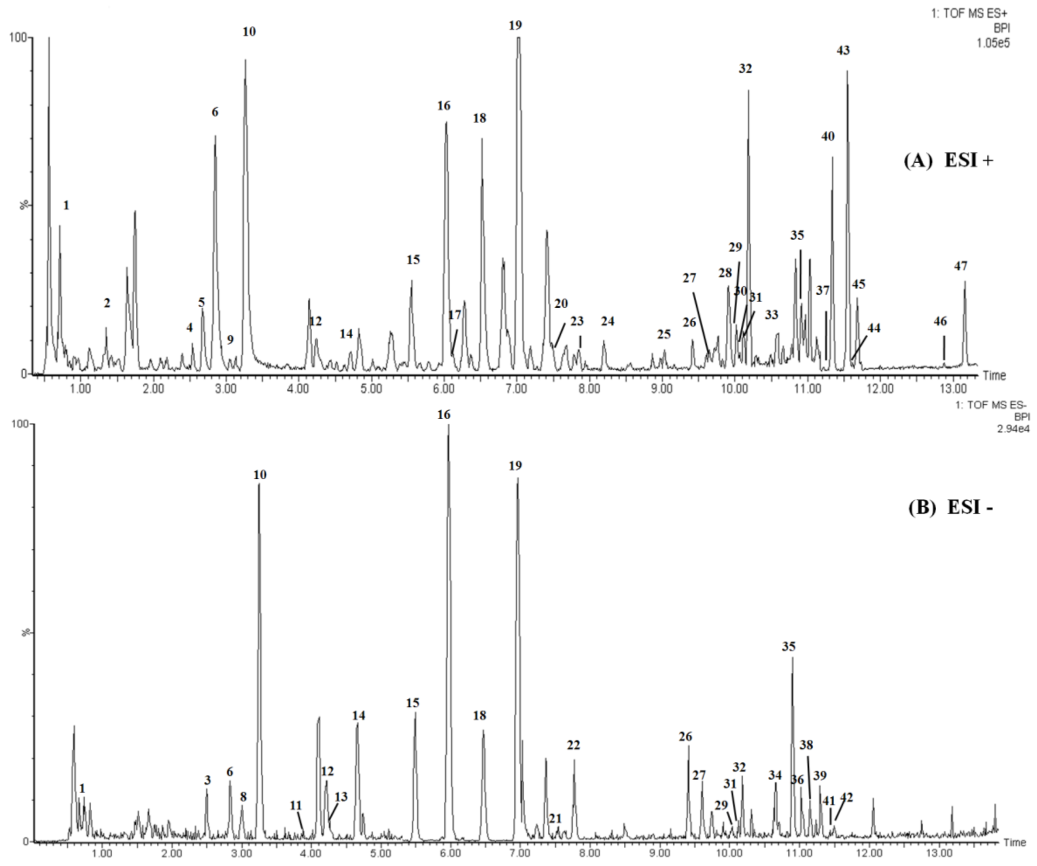

Figure S4: The chemical profiles of CSGS extract by UPLC-Q-TOF/MS in positive ESI mode (A) and negative ESI mode (B).

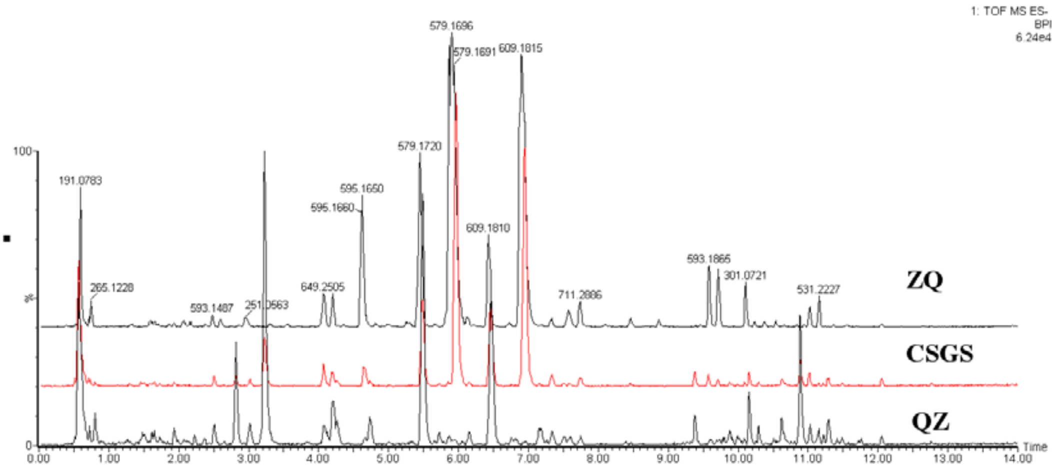

Figure S5: The BPI chromatograms of the extracts of CSGS, QZ and Zhi-Qiao in negative mode.
